# Rare adverse root development after regenerative endodontic procedures: two case reports and contributing factors analysis

**DOI:** 10.3389/froh.2026.1844033

**Published:** 2026-06-09

**Authors:** Huasheng Xu, Lu Liu, Beibei Xie, Xiaoyi Zhong

**Affiliations:** 1 Stomatology Department, Minzu Hospital of Guangxi Zhuang Autonomous Region, Nanning, China; 2 Conservative Dentistry & Endodontics Department, College of Stomatology, Guangxi Medical University, Nanning, China; 3 Comprehensive Care Clinic, College of Stomatology, Guangxi Medical University, Nanning, China

**Keywords:** case report, external force, infection, regenerative endodontic procedures, unusual root development

## Abstract

Regenerative endodontic procedures (REPs) promote continued root development in immature teeth with pulp necrosis. However, endodontic infection or physical trauma during tooth development may lead to abnormal root formation. This report describes two cases of immature teeth with periapical disease secondary to dens evaginatus, both treated with REPs and followed up for 4 years. Uncommon root developments, including root curvature and segmental root formation, were observed. A review of the relevant literature was conducted to identify the potential contributing factors. Clinicians should be aware that even clinically successful REPs may result in unusual root morphology, especially in cases with pre-existing inflammation or concurrent orthodontic treatments.

## 1 Introduction

Immature permanent teeth are characterized by open apices, weak canal walls, and discordant crown-to-root ratios. Traditional treatment for immature teeth with pulp necrosis involves apexification or the creation of an apical barrier using mineral trioxide aggregate (MTA). However, long-term calcium hydroxide dressing is the classic approach for apexification, which induces an apical barrier but requires multiple visits over many months and carries a risk of root fracture (1). MTA was subsequently introduced as an apical barrier technique, offering a more predictable outcome and a shorter treatment course compared with apexification (2). However, these therapies do not promote root development, as they rely on hard tissue barrier formation rather than biological regeneration (3).

With advances in tissue engineering, regenerative endodontic procedures (REPs) based on stem cells, growth factors, and scaffolds have been recommended by the American Association of Endodontists (AAE) (4) as the primary therapy for immature teeth with pulp necrosis. Increasing evidence has indicated that REPs can promote continued root development, leading to significant increases in root length and the thickening of dentinal walls (5). Despite these satisfactory outcomes, the final root morphology may be unpredictable (6). Chen et al. identified five radiographic outcomes following REPs: increased root canal wall thickness, root maturation with apex closure, root development with an open apex, severe intracanal calcification (7), and the formation of a hard tissue barrier between the coronal and apical portions (8). In this report, we describe two unusual types of root development with root canal curvatures and separate root segments following REPs, and explore the potential contributing factors.

## 2 Case description

All case reports were prepared in accordance with the CARE guidelines. The PRICE 2020 guidelines were consulted during the manuscript preparation (9). The timelines for Case 1 and Case 2 are summarized in Tables 1, 2.

**Table 1:** Time points for Case 1.

Time point	Initial visit	3 weeks	3 months	12 months	19 months	29 months	4 years
Event	Treatment plan (REPs performed)	Sinus tract resolved	Asymptomatic periapical lesion resolved	Continued root development	Root curvature began	Form a branch-like structure	Complete root development; intracanal calcification

**Table 2:** Time points for Case 2.

Time point	Initial visit	3 weeks	3 months	6 months	12 months	2 and 3 years	4 years
event	Treatment plan (REPs)	Start of periapical healing	Asymptomatic; resolution of the periapical lesion	Calcified barrier	Root length increased; intracanal calcification	Progressive calcification	Form a separate root tip

### 2.1 Case 1

A 9-year-old girl, accompanied by her mother, was referred for the treatment of the mandibular right second premolar (tooth #45). Her mother reported that the patient had been suffering from recurrent pain and gingival swelling for a month. The pain was spontaneous, moderate, and constant. The patient's medical history was non-contributory, and she had no known drug allergies. No relevant family or psychosocial history was noted, and the patient had not undergone any prior dental interventions for this tooth.

The clinical examination revealed a fractured dens evaginatus in the central fossa of tooth #45 (Figure 1A). The buccal vestibule exhibited swelling, with a traceable sinus tract (Figure 1B). The tooth exhibited moderate tenderness to percussion and biting. The thermal and electric pulp vitality tests yielded negative responses. The periodontal probing depth and mobility were within the normal limits. Periapical radiography revealed a wide-open apex and periapical radiolucency, with the sinus tract tracing to the apex (Figure 1C). Based on the clinical and radiographic findings, the presumptive diagnosis was pulp necrosis with a chronic apical abscess secondary to a fractured dens evaginatus. The prognosis was favorable owing to the patient's young age and the presence of an open apex. The differential diagnoses included periapical periodontitis and acute apical abscess. No significant diagnostic challenges were encountered, and the final diagnosis for this case was a chronic apical abscess.

**Figure 1:**
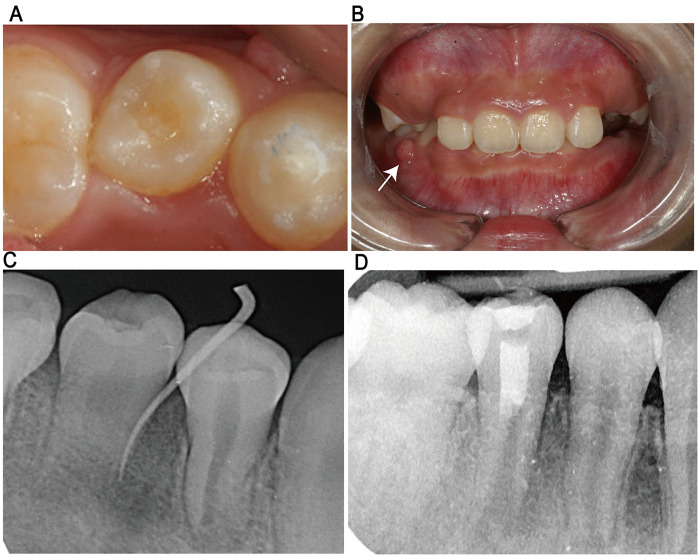
**(A)** Fractured dens evaginatus (central cusp). **(B)** Sinus tract located on the buccal mucosa of tooth #45 (arrow). **(C)** Radiograph showing that the gutta-percha is directed to the apex of tooth #45 after tracing the sinus tract and periapical lesion. **(D)** Immediate postoperative radiograph.

Treatment options, including non-surgical apexification, extraction, REPs, MTA apical barrier, and no treatment, were discussed with the patient's guardian. REPs were selected to promote continued root development, and informed consent was obtained. After administering local anesthesia with 2% lidocaine (Tiansheng Pharmaceutical, China) without a vasoconstrictor, the tooth was isolated using a rubber dam (Henry Schein, USA). An access cavity was prepared, and the pulp chamber was irrigated with 1.5% sodium hypochlorite (NaOCl) (Longly Biotechnology, China) to minimize contamination of the apical region. The working length (WL) was determined using an electronic apex locator combined with a periapical radiograph, and the canal was gently irrigated with 20 mL of 1.5% NaOCl using a side-vented needle placed 1 mm short of the WL. The canal was then flooded with 20 mL of 17% ethylenediaminetetraacetic acid (EDTA) (Longly Biotechnology, China), followed by physiological saline. After drying with paper points, a double-antibiotic paste (equal parts of ciprofloxacin and metronidazole) was placed as an intracanal medicament. The cavity was temporarily sealed with glass ionomer cement (GIC) (Fuji IX GP, Japan).

Two weeks later, the patient was asymptomatic, and the sinus tract had healed. After anesthesia and rubber dam isolation, the temporary filling was removed, and the canal was irrigated with 17% EDTA followed by saline. Bleeding was induced by over-instrumentation with a #25 handle K file and allowed to form a blood clot below the cementoenamel junction. White MTA (PROROOT, Dentsply, USA) was placed over the clot, and a wet cotton pellet was applied to facilitate setting. The cavity was sealed again with GIC, and a postoperative radiograph was obtained (Figure 1D). At a subsequent visit, the access cavity was restored with composite resin (3M ESPE, USA) after confirming MTA setting, using a self-etch adhesive system (Single Bond Universal 8, 3M ESPE), as recommended by Shin et al. (10), who demonstrated that a one-step self-etch adhesive provides optimal bonding to ProRoot MTA (WMTA) (Dentsply, Tulsa, OK, USA) without prior phosphoric acid etching.

At the 3-month follow-up, the patient was asymptomatic, and radiography revealed a complete resolution of the periapical radiolucency (Figure 2A). However, coronal discoloration was observed (Figure 2H). At 6 and 12 months (Figures 2B,C), radiographs revealed an increase in root length and dentine wall thickness, accompanied by a decrease in apical diameter. The results of the electricity pulp tests remained negative.

**Figure 2:**
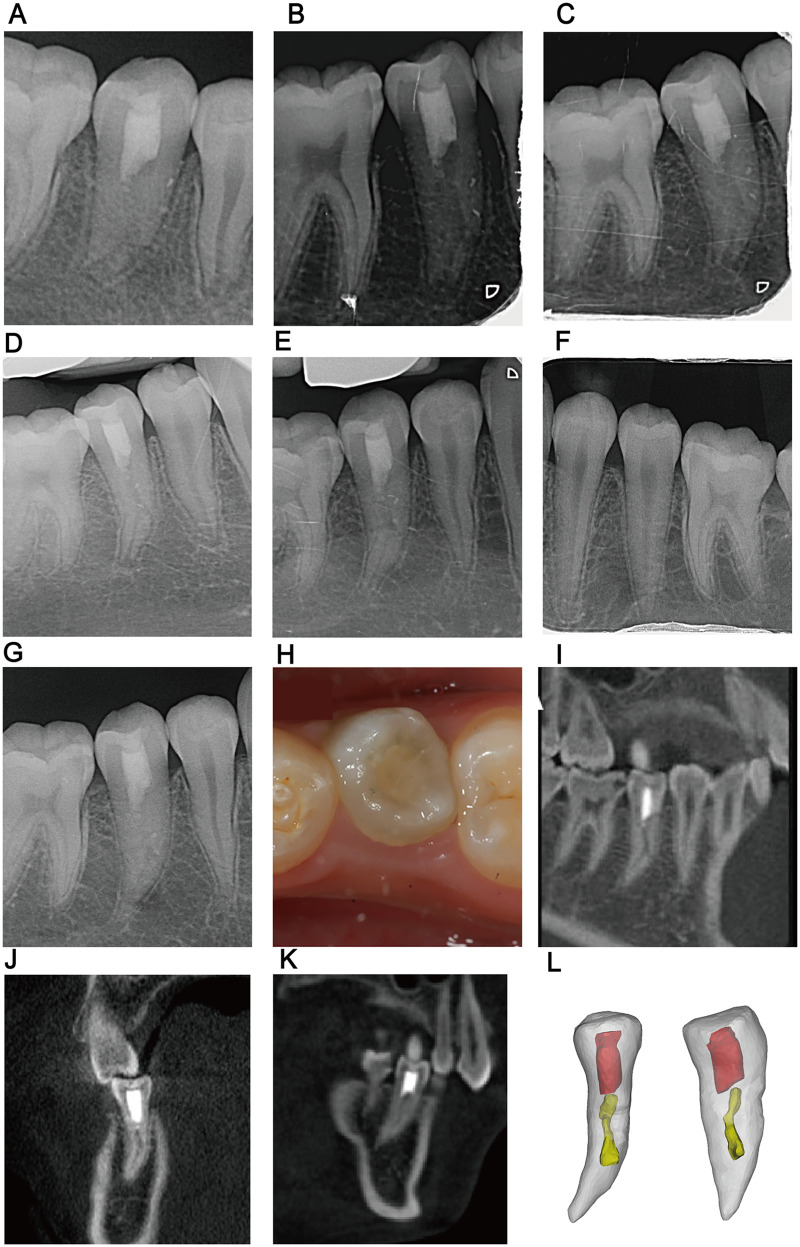
**(A)** Initial resolution of periapical radiolucency and hard tissue bridging (at 3-month). **(B)** Complete healing of the periapical lesion (at 6-month). **(C)** Continuous development of the root and canal branch begins to form (at 12-month). **(D,E)** Progressive root curvature (at 19- and 29-month). **(F)** Contralateral tooth. **(G)** The ambiguous root canal with moderate bending root (at 4 years). **(H)** Discoloration of teeth after REPs. **(I,J)** CBCT images of the root canal at the 4-year follow-up. **(K,L)** CBCT and 3D reconstruction of root and canal morphologies at the 6-year follow-up.

At 19 and 29 months (Figures 2D,E), continued root development was observed, with the root exhibiting progressive curvature, whereas the contralateral tooth remained straight (Figure 2F). A radiopaque, branch-like structure was observed in the middle third of the root. The tooth began to show a weak positive response to the thermal and electricity pulp test (EPT) tests.

At the 4-year follow-up, the tooth responded positively to both thermal and electric pulp tests. Periapical radiography revealed complete root development and advanced intracanal calcification (Figure 2G). Cone-beam computed tomography (CBCT) confirmed partial canal obliteration with a curved but patent canal and increased wall thickness (Figures 2H,I). The CBCT images and 3D reconstruction at the 6-year follow-up also showed the curve and irregular root canal morphology (Figures 2K,L).

### 2.2 Case 2

An 11-year-old girl was referred by her orthodontist for an evaluation of her mandibular left second premolar (tooth #35), which showed periapical radiolucency on routine radiography (Figure 3A). The patient reported moderate pain and slight swelling in the area. Orthodontic treatment was initiated 2 years earlier. Her medical history and family history were non-contributory.

**Figure 3:**
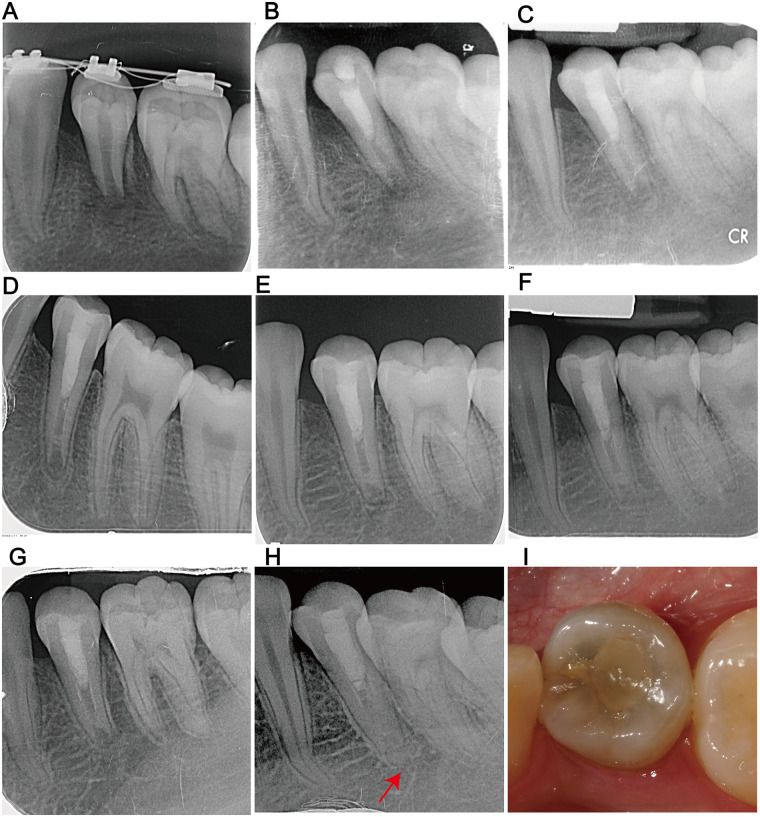
Periapical images during the 4-year follow-up period. **(A)** Preoperative periapical radiograph of tooth# 35 showing apical radiolucency, an open apex, and a thin canal wall. **(B)** Immediate postoperative radiograph. **(C)** Complete resolution of the periapical lesion (at 3-month). **(D)** Calcification barrier formation in the canal (at 6-month). **(E)** Increase in the root length and level of calcification (at 1-year). **(F,G)** Further and progressive calcification in the body of the root (at 2-3 years). **(H)** Separate root detached from the main root, and extensive calcification was observed in the apex, which seems to indicate apex closure (arrow) (at 4 years). **(I)** Discoloration of tooth #35 at the 6-month recall.

Clinical examination revealed a fractured dens evaginatus and a buccal sinus tract associated with tooth #35. The tooth was sensitive to percussion and palpation without periodontal pocketing. Both thermal and EPT tests were negative. Radiography revealed an open apex, thin dentine walls, and a periapical lesion. The differential diagnosis considered orthodontic-induced apical pathology versus dens evaginatus-related necrosis. The presence of a fractured dens evaginatus supported the latter. Presumptive diagnosis: pulp necrosis with chronic apical abscess secondary to dens evaginatus. The final diagnosis was a chronic apical abscess without any diagnostic challenges.

After discussion with the patient and her guardian, informed consent was obtained, and REPs were performed as described in Case 1. At the third visit (21 days after the initial treatment), clinical examination showed resolution of the sinus tract, and the periapical radiograph exhibited the start of periapical healing with a decrease in the size of the radiolucent lesion (Figure 3B).

At 3 months, the patient was asymptomatic, and radiography indicated the resolution of the periapical lesion (Figure 3C). At 6 months, a calcified barrier was visible in the canal (Figure 3D). Coronal discoloration was observed (Figure 3I). At 1 year, the root length had increased and intracanal calcification had progressed, while the dentine wall thickness showed minimal change (Figure 3E). The pulp tests remained negative.

At 2 and 3 years, progressive calcification was observed with no significant change in root morphology (Figures 3F,G). At 4 years, radiography revealed a separate radiopaque structure detached from the main root, resembling a distinct root tip. Extensive calcification was observed in the apical portion of the main root (Figure 3H). The patient remained asymptomatic, and the pulp tests were negative.

### 2.3 Prognosis

 Given the patient's young age, absence of systemic disease, and the availability of REPs as a biologically based treatment option, the prognosis is favorable. Intervention adherence and tolerability: Both patients attended all scheduled follow-up visits and reported no discomfort or difficulty in complying with the treatment protocol.

## 3 Discussion

The “AAE Clinical Considerations for Regenerative Endodontic Procedures (Revised 2021)” defines the success of REPs through three goals: resolution of clinical symptoms, the healing of periapical bones, and the continued root development (11). In the cases presented above, clinical symptoms resolved, and periapical healing and continued root development were achieved. However, both cases exhibited unusual root development, including root curvatures and separate root segments. These findings have potential clinical implications, such as an increased risk of root fracture or complexity in future endodontic retreatment.

Recent evidence indicates that the etiology of pulp necrosis may affect the outcomes of REPs. A study utilizing bioinformatic analysis revealed that the type of dentoalveolar trauma affects the molecular pathways activated during regeneration, with differential expression of genes, such as *TNF-*α, *FN1*, and *FGF2*, potentially conditioning the success of treatment (12). This provides a mechanistic basis for understanding why teeth with similar clinical presentations—such as our two cases, both secondary to dens evaginatus—may exhibit divergent regenerative outcomes.

The mechanism underlying unusual root development remains unclear, but several factors, including infection, severe trauma, external orthodontic force, and iatrogenic causes, have been implicated. Hertwig's epithelial root sheath (HERS) (13) and mesenchymal stem cells from the apical papilla (SCAP) (14) play critical roles in root formation, including determining root shape, promoting dentinogenesis, and facilitating cementum deposition (15). A comprehensive review by Wang et al. elucidated the signal pathways critical for tooth root formation, identifying nuclear factor I C (NFIC) as a key transcriptional factor that specifically controls root dentin formation independent of crown development. Disruption of NFIC-dependent pathways can lead to root anomalies without affecting the crown morphology (16). This molecular framework helps explain how localized disruption, whether by inflammation or mechanical force, can produce root-specific abnormalities, as observed in our cases, while crown development remains unaffected. Disruption of HERS or SCAP due to trauma, infection, or mechanical forces may lead to aberrant root development (17).

In Case 2, orthodontic treatment was ongoing during the REPs follow-up. Orthodontic forces may have mechanically separated HERS from the developing root tip, leading to the formation of a detached root segment. This phenomenon is supported by a case report documenting unusual root development with discontinuity in the middle third region following surgical repositioning (18). Despite the physical separation of the coronal and apical segments, the tooth remained functional, demonstrating the remarkable recovery capacity of HERS even after mechanical disruption.

Experimental studies suggest that SCAP and HERS survive inflammation and contribute to root formation (19). However, severe or persistent inflammation can alter the local microenvironment, impair stem cell differentiation, and result in irregular root morphology. In Case 1, the presence of long-standing periapical inflammation may have disrupted HERS integrity, thereby contributing to the development of root curvature. Epithelial–mesenchymal interactions between HERS and SCAP are essential for guided root growth, and disruption of this interface may lead to uncontrolled or deviated root development (20).

Emerging molecular evidence provides deeper insights into how inflammation affects SCAP function. A study demonstrated that in apical periodontitis, SCAPs exhibit decreased expression of METTL3, an m6A methylase that regulates odontogenic differentiation via NFIC. Overexpression of METTL3 restored the odontogenic potential of inflamed SCAPs and suppressed the inflammatory cytokines TNF-α and IL-6 (21). Similarly, Huang et al. identified fat mass and obesity-associated protein (FTO) as another critical regulator of SCAP odontoblastic differentiation under inflammatory conditions. Their study showed that lipopolysaccharide-induced inflammation downregulates FTO expression, impairing differentiation via the SMOC2 pathway. Importantly, the overexpression of FTO partially counteracted the inhibitory effects of inflammation (22). Collectively, these findings suggest that the inflammatory microenvironment disrupts SCAP function through multiple epigenetic and molecular mechanisms, potentially contributing to aberrant root morphology. This epigenetic mechanism helps explain why prolonged inflammation may lead to unusual root development, as observed in Case 1.

These cases highlight the importance of considering external forces and inflammatory conditions when planning the REP. Orthodontic treatment during the regenerative phase may pose a risk for unusual root development (23). Clinicians should consider delaying orthodontic tooth movement until the completion of root maturation. Long-term follow-up is essential to monitor such adverse outcomes.

This study was limited by the small number of cases and the lack of histological confirmation. The exact timing and magnitude of the orthodontic forces in Case 2 were not recorded, limiting causal inference. Further studies with larger cohorts and standardized protocols are needed to better understand the factors influencing root development after REPs. Based on these findings, we suggest that orthodontic treatment be postponed until at least 2 years after REPs and that clinicians document any history of preoperative sinus tract duration as a potential risk factor for aberrant root development.

## 4 Conclusions

This case series describes two rare root developments following REPs, including root curvature and segmental root formation. These outcomes may be associated with periapical inflammation and external mechanical forces, such as orthodontic tooth movement. These findings align with emerging evidence that inflammatory microenvironments disrupt SCAP function via epigenetic mechanisms, potentially contributing to aberrant root morphology. Clinicians should be aware of these risks and consider delaying orthodontic treatment during REPs. Both teeth remained functional and asymptomatic at 4 years, demonstrating that even suboptimal radiographic outcomes can coexist with clinical success (24). This suggests that unusual root morphology does not necessarily compromise clinical success.

## Data Availability

The original contributions presented in the study are included in the article/Supplementary Material, further inquiries can be directed to the corresponding author.
